# Biliary hyperkinesia, a new diagnosis or misunderstood pathophysiology of dyskinesia: A case report

**DOI:** 10.1016/j.ijscr.2019.01.011

**Published:** 2019-01-19

**Authors:** John A. Bates, Kelly Dinnan, Victoria Sharp

**Affiliations:** Beaumont Health Farmington Hills, General Surgery Department, 28050 Grand River Avenue, Farmington Hills, MI 48336, USA

**Keywords:** Biliary hyperkinesia, Normokinetic biliary dyskinesia, Biliary dyskinesia, Case report

## Abstract

**Introduction:**

Biliary colic, characterized by intermittent right upper quadrant abdominal pain is a common complaint in the United States population. Patients whose pain is undiagnosed by ultrasound generally undergo hepatobiliary iminodiacetic acid scan with cholecystokinin stimulation (HIDA-CCK) to assess function of the gallbladder and biliary tree. Traditionally, two outcomes are possible based on a measured ejection fraction of the gallbladder: either dyskinesia or normal function is diagnosed. Biliary dyskinesia, or hypokinesia of the gallbladder, is accepted as an ejection fraction less than 35%, while an accepted normal functioning gallbladder ejection fraction is greater than 35%.

**Case:**

We report a case of a fifteen-year-old female who had functional gallbladder disease per Rome IV criteria due to intermittent biliary colic, with exception to the ejection fraction measurement which was elevated at 96.5%. She underwent laparoscopic cholecystectomy with complete symptom resolution.

**Discussion:**

As demonstrated in the literature reviewed here, these subsets of patients, who present with normal to high ejection fractions, have undergone laparoscopic cholecystectomy with resolution of pain in several case studies.

**Conclusion:**

Many unknown variables still exist due to lack of prospective studies, most notably the pathophysiology and definitive indications for surgical treatment. As such, we propose that surgical options should not be limited to those who display the traditional findings of biliary dyskinesia, but also patients who demonstrate typical symptoms with normal to elevated ejection fraction, following work up to rule out the extensive differential diagnoses for right upper quadrant abdominal pain.

## Highlights

• Bilairy hyperkinesia or normokinetic biliary dyskinesia may have no “pathological” findings of disease or dysfunction.
• The majority of patients, both adult and pediatric, have symptoms improved/resolved following laparoscopic cholecystectomy.
• Multiple case series have been performed, and no conclusive pathophysiological abnormality has been observed.
• The literature would benefit from a larger prospective study.

## 1 Introduction

Biliary colic, characterized by intermittent right upper quadrant (RUQ) abdominal pain is a common complaint in the United States population. Patients whose pain is undiagnosed by ultrasound generally undergo hepatobiliary iminodiacetic acid scan with cholecystokinin stimulation (HIDA-CCK) to assess function of the gallbladder and biliary tree. Traditionally, two outcomes are possible based on a measured ejection fraction of the gallbladder: either dyskinesia or normal function is diagnosed. Biliary dyskinesia, or hypokinesia of the gallbladder, is accepted as an ejection fraction less than 35%, while an accepted normal functioning gallbladder ejection fraction is greater than 35%.

An increasing number of patients undergo evaluation for RUQ abdominal pain traditionally consistent with gallbladder disease but imaging findings are unremarkable. These patients may benefit from cholecystectomy, with many obtaining complete resolution of symptoms if an elevated ejection fraction is found on HIDA-CCK.

## 2 Case report

A fifteen year old Caucasian female (BMI 25 kg/m^2^) was sent to the surgical office by her primary care physician for a one-month history of increasingly intermittent, right upper quadrant, colicky abdominal pain that radiated to the back. She experienced the onset of pain within 15–20 min following a meal and it spontaneously resolved in thirty minutes. She reported nausea when pain is most severe, but otherwise denied further symptoms. Further history was noncontributory with exception of her mother and sister requiring cholecystectomy at a similar age. The abdominal ultrasound obtained from an outpatient imaging center reported no cholelithiasis, wall thickening, murphy’s sign, and a common bile duct measuring at 3.6 mm. Physical exam in office was unremarkable, noting no jaundice or pallor. Abdominal exam revealed scaphoid, soft abdomen, without mass or explanation/etiology for postprandial abdominal pain. After further discussion with the patient and her mother, she was sent for a HIDA -CCK to evaluate for biliary dyskinesia.

**Fig. 1:**
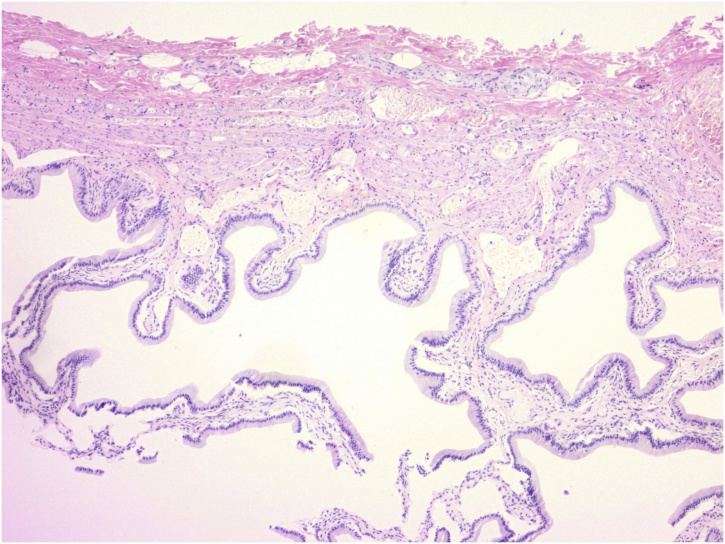
Gallbladder Wall at 40× without visible inflammation or wall thickening.

**Fig. 2:**
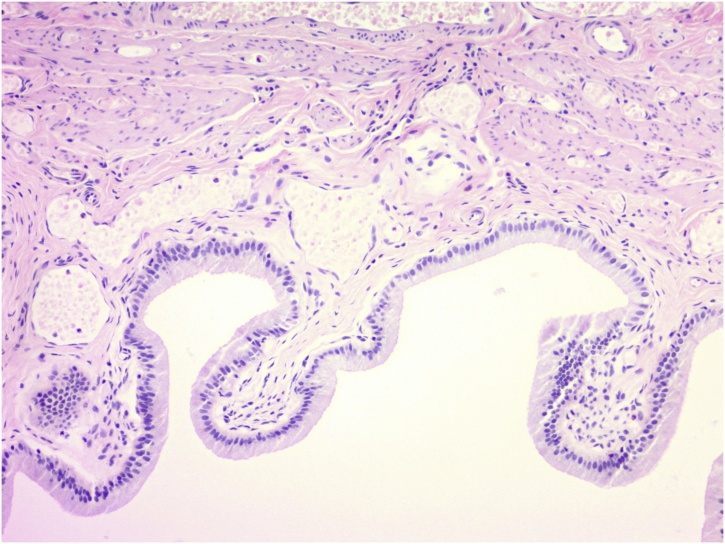
Gallbladder Wall at 100× without visible inflammation or wall thickening.

The patient returned to the office the following week with HIDA revealing a patent cystic and common bile ducts without evidence of acute cholecystitis. The patient’s ejection fraction was measured to be 96.5% following CCK administration. Ultrasonographer report stated the patient exhibited no reproduction in symptoms during infusion of CCK. We discussed findings with the patient and the decision was made to perform esophagogastroduodenoscopy (EGD) with biliary crystal analysis to exclude microlithiasis, gastritis, or peptic ulcer disease as the etiology of her symptoms. EGD was performed with gastric antral biopsies and bile collection. Pathology revealed no significant inflammation, intestinal metaplasia, dysplasia, or malignancy. Biliary crystal analysis was negative for monosodium urate or calcium pyrophosphate crystals. One-month trial of a proton pump inhibitor with a gastroesophageal reflux (GERD) minimizing diet performed without symptomatic relief. The patient returned to the office where ROME criteria for Irritable Bowel Syndrome (IBS) were ruled out and ROME IV criteria for biliary dyskinesia reviewed (Table 1) [1]. Patient was then taken for an elective cholecystectomy. The procedure was without complication and grossly the gallbladder appeared non- inflamed, dilated, or hydropic. Pathological exam as seen in Fig. 1, Fig. 2, returned a normal gallbladder wall without thickening or inflammation.

**Table 1:** Rome IV Criteria [1].

*Functional gallbladder and sphincter of Oddi disorders*
1. Biliary pain
2. Absence of gallstones or other structural pathology
3. Is located in the epigastrium and/or right upper quadrant
4. Occurs at variable intervals (not daily)
5. Lasts at least 30 min
6. Builds up to a steady level
7. Is severe enough to interrupt daily activities or lead to an emergency department visit
8. Is not significantly (<20 percent) relieved by bowel movements, postural changes, or acid suppression
*In addition, the criteria that are supportive of functional gallbladder disorder, but are not required, include*:
9. Low ejection fraction on scintigraphy
10. Normal liver enzymes, conjugated bilirubin, and amylase/lipase

Postoperatively the patient reports she is now pain-free, tolerating all foods without reproduction of symptoms. Three month telephone follow up finds that the patient continues to be pain free without symptom recurrence.

## 3 Literature review

After obtaining parental consent for publication and exemption from IRB review as it is a single case study, an electronic literature review demonstrated few articles have been published on findings of biliary hyperkinesia as a diagnosis for right upper quadrant colicky abdominal pain. This review has been reported in line with the SCARE criteria [2]. It appears the first report was published in 1999 by Cook et al. at Ohio State University. It was a retrospective study of fourteen patients, seven of whom were managed non-operatively and even with laparoscopic cholecystectomy. No significant difference between age, ejection fracture, symptoms, or follow up in groups was established and the study found 100% symptomatic relief with cholecystectomy and no symptomatic relief in patients who underwent non-operative therapy. They concluded that hyperkinesis of the gallbladder responds favorably to surgical therapy [3].

Another Case series of the patients by Huckaby et al. at Saint Christopher’s Hospital for Children, in 2013, was focused on adolescent biliary colic with hyperkinesia and no apparent gallbladder disease on imaging or laboratory findings. Workup of these patients revealed an average ejection fraction of 76% with symptomatic relief following laparoscopic cholecystectomy. Mean follow up of these patients post-operatively was 14 months [4]. Gallbladder pathology on two of the three revealed chronic inflammation.

Currently James Greenberg at Mercy Walworth, Lake Geneva, Wisconsin published the largest case series in 2018. This was a series of thirteen patients with an average ejection fraction of 90.5%. The patient age range was 12–75 years old. Pathology revealed chronic cholecystitis in all but two patients and all patients had complete symptom relief with follow up, done between three months and three years [5].

## 4 Discussion

Biliary Dyskinesia is a widely accepted and common indication for cholecystectomy in the United States. Recent literature shows increasing laparoscopic cholecystectomies for biliary dyskinesia especially in the pediatric population, likely due to multiple factors including the Western diet, increasing rates of childhood obesity and sedentary lifestyles [6]. The widely accepted diagnosis of biliary dyskinesia is comprised of vague right upper quadrant pain with absence of gallstones or gallbladder wall thickening and an abnormally low gallbladder ejection fraction on hepatobiliary iminodiacetic acid (HIDA) scan. The threshold for” low” ejection fraction, commonly accepted as 35% following infusion of cholecystokinin (CCK) analog, was originally accepted after a publication by Fink-Bennett et al. in 1991 [7]. Biliary dyskinesia has traditionally been a diagnosis of exclusion requiring workup to rule out an extensive differential of other gastric and hepatobiliary causes of pain including gastritis, peptic ulcer disease, sphincter of oddi dysfunction, inflammatory bowel disorders, and cholecystitis [8,9]. This workup requires a focused history and physical, as well as ultrasonographic evaluation of the liver, gallbladder, and biliary tree. If the work up fails to establish a diagnosis, further testing is then undertaken which may include an EGD and/or HIDA scan. For a large percentage of the population this is sufficient enough to establish a diagnosis with a defined treatment path. For a small fraction of patients this testing may exclude all previously held differential diagnoses.

Biliary Hyperkinesia, first described in the literature in 1999, is similar to biliary dyskinesia in that both are diagnoses of exclusion [3]. Following traditional evaluation for gallbladder pathology/dysfunction, no abnormality is seen except an elevated ejection fraction on HIDA. Diagnostic ejection fraction for biliary hyperkinesia varies per publication anywhere from 65% to greater than 90% [[3], [4], [5]]. In our case, the patient was found to have an ejection fraction of 96.5% putting it within the range of all previous studies. Interestingly, our patient did not exhibit reproduction of symptoms with cholecystokinin administration, which also varied among the studies reviewed.

Further diagnostic evaluation also varies or is not reported in previous publications. Some studies report an EGD was performed but none with collection of bile for crystal evaluation to rule out microlithiasis are reviewed. On limited abdominal ultrasound the common bile duct diameters for evaluation of possible sphincter of oddi dysfunction are not mentioned or evaluated on the Milwaukee classification system. Intraoperatively our patient had no signs of chronic inflammation or other abnormality and pathology revealed a normal gallbladder without inflammation. Pathological findings also varied between series from normal to chronic cholecystitis or inflammation, though no correlation was seen between pathological findings and post-operative symptom relief. Previous authors speculated that the chronic cholecystitis found on pathology could represent mucosal injury from increased intraluminal pressure due to cholecystokinin hypersensitivity [4]. Though this cannot be discounted, in our patient and several patients in previous studies, many of the specimens did not reveal chronic inflammatory changes. This may suggest different stages or chronicity of disease but this again does not correlate with chronicity of symptoms or age.

The varied findings both on imaging and pathological examination raise more questions than they answer regarding the pathophysiology of this disease. The few published cases differ, but nearly all patients report symptomatic relief postoperatively (Table 2). One notable retrospective study by Ducoin et al. retrospectively reviewed patients with “Normokinetic biliary dyskinesia” who’s ejection fraction was 75.1 ± 19.4% which falls within previous studies of hyperkinesia. In this specific study seventeen of nineteen patients had complete resolution of symptoms following laparoscopic cholecystectomy [10]. Unfortunately data was not provided to evaluate the ejection fraction from the patients without complete resolution of symptoms for comparison.

**Table 2:** Biliary Hyperkinesia Case Study Comparison.

	n	Mean Age	Avg EF	Pain w/CCK (%)	Chronic inflammation (%)	Symptom Relief S/P Chole (%)
Bates et al.	1	15	98.5	0	0	100
Ducoin et al. [10]	19	48.4	75.1	100	94.7	89.4
Greenberg [5]	13	43	90.5	84.6	84.6	84.6
Huckaby et al. [4]	3	15.7	73.3	33.3	66.7	100

But, what of the small proportion of patients who have all the symptomatic indications of biliary dyskinesia without hypokinesia indications on imaging studies? As seen in previous studies, symptomatic relief is successfully obtained with laparoscopic cholecystectomy. The accepted symptom resolution in biliary dyskinesia is 94–100% [11,12]. Symptomatic relief of hyperkinesis studies in Table 2 reveals a cure rate similar to that expected of biliary dyskinesia. Multiple studies have been performed showing symptomatic relief is more highly correlated to lower ejection fraction [13,14], while others directly

refute this showing ejection fraction is not a predictor of symptomatic outcome [15,16]. We propose that based on the aforementioned studies, including our own case experience, that the decision to proceed with laparoscopic cholecystectomy should not be ruled out based on an ejection fraction that does not fall within the less than 35% category, and that those with hyperdynamic ejection fractions may well benefit from gallbladder removal for symptom relief.

## 5 Conclusion

Multiple well-documented case studies and series have shown that the majority of patients with the established diagnosis of biliary dyskinesia benefit from laparoscopic cholecystectomy. Though the pathophysiology of this disease is still debated, the symptomatic relief of biliary colic continues to be evident in the United States following surgery with HIDA proven biliary dyskinesia. Why could the pathophysiology not also affect individuals with ejection fraction greater than 35% if studies cannot agree whether the numeric value of ejection fraction correlates with symptoms? Multiple case reports have been published in the literature of patients experiencing symptomatic relief following cholecystectomy with HIDA ejection fractions greater than 35%, or normal ejection fraction.

Admittedly this topic continues to require extensive study and clarification. Normokinetic biliary dyskinesia and biliary hyperkinesia remain poorly studied and misunderstood diagnoses in the literature. Differences in the few case studies reveal several similarities including typical biliary colic symptoms, normal to high ejection fraction, and symptomatic relief following cholecystectomy. Many unknown variables still exist due to lack of prospective randomized control studies, most notably the pathophysiology and definitive indications for surgical treatment. Table 2 reveals both pediatric and adult patients whose symptoms improved or resolved following cholecystectomy. As such, we propose that surgical options should not be limited to those who display the traditional findings of biliary dyskinesia, but also patients who demonstrate typical symptoms with normal to elevated ejection fraction, following work up to rule out the extensive differential diagnoses for right upper quadrant abdominal pain.

## Conflict of interest

None.

## Funding

None.

## Ethical approval

IRB Approval obtained from Beaumont Health Systems IRB review board.

## Consent

Written informed consent was obtained from parental guardian.

## Author’s contribution

John A Bates, DO: Primary author, Corresponding author, study concept or design, data collection, data analysis or interpretation, writing the paper.

Kelly Dinnan, DO: Guarantor, contributing author. Parental consent obtained and IRB approval.

Victoria Sharp, DO: Contributing author, review.

## Registration of research studies

Retrospective review of case without identification or new/investigational treatments.

## Guarantor

Kelly Dinnan, DO.

## Provenance and peer review

Not commissioned, externally peer-reviewed.
